# Поиск новых иммуногистохимических и циркулирующих маркеров инсулиномы

**DOI:** 10.14341/probl13466

**Published:** 2024-05-13

**Authors:** М. Ю. Юкина, Е. А. Трошина, Л. С. Урусова, Н. Ф. Нуралиева, Л. В. Никанкина, В. А. Иоутси, О. Ю. Реброва, Н. Г. Мокрышева

**Affiliations:** Национальный медицинский исследовательский центр эндокринологии; Национальный медицинский исследовательский центр эндокринологии; Национальный медицинский исследовательский центр эндокринологии; Национальный медицинский исследовательский центр эндокринологии; Национальный медицинский исследовательский центр эндокринологии; Национальный медицинский исследовательский центр эндокринологии; Национальный медицинский исследовательский центр эндокринологии; Российский национальный исследовательский медицинский университет имени Н.И. Пирогова; Национальный медицинский исследовательский центр эндокринологии

**Keywords:** инсулинома, иммуногистохимические маркеры, циркулирующие маркеры

## Abstract

ОБОСНОВАНИЕ. Инсулинома — нейроэндокринная опухоль, основным проявлением которой является гипогликемия. Однако симптомы гипогликемии длительно могут носить неспецифический характер, особенно вне провокационных условий, и нередко опухоль манифестирует с жизнеугрожающего состояния — гипогликемической комы. В связи с этим своевременная лабораторная диагностика инсулиномы и определение ее агрессивного течения являются одним из приоритетных направлений в современных исследованиях.ЦЕЛЬ. Поиск новых иммуногистохимических (ИГХ) и циркулирующих маркеров (ЦМ) инсулиномы, в том числе ее агрессивного течения.МАТЕРИАЛЫ И МЕТОДЫ. Включены пациенты, обследованные в ФГБУ «НМИЦ эндокринологии» в период 2017–2022 гг. и прооперированные по поводу инсулин-продуцирующей опухоли. Перед хирургическим вмешательством и через 2–12 месяцев после него выполнен забор крови с определением таргетных белков-маркеров. Некоторым пациентам проведено расширенное ИГХ исследование опухоли, окружающей ткани, и островков Лангерганса с первичными антителами к таргетным белкам-маркерам с оценкой степени их экспрессии. Для определения агрессивного течения опухоли были охарактеризованы по степени злокачественности (Grade), количеству новообразований и признакам рецидива.РЕЗУЛЬТАТЫ. На основании анализа литературы и патогенетических характеристик инсулиномы выбраны следующие кандидаты для таргетных белков-маркеров: кокаин- и амфетаминрегулируемый транскрипт (CART), хромогранин В (ХрВ), нейроэндокринный секреторный протеин 55 (NESP55), глюкагоноподобный пептид 1 (ГПП1), арилалкиламин-N-ацетилтрансфераза (AA-NAT), мелатонин и, исключительно для ИГХ исследования, протеин D52 (TPD52), а также рецепторы к глюкагоноподобному пептиду-1 (рГПП1) и мелатонину (MTNR1b). В исследование включен 41 пациент, из них 10 проведено расширенное ИГХ исследование. У пациентов, как с агрессивной, так и неагрессивной инсулиномой, после хирургического лечения уровни ЦМ статистически значимо не менялись и у отдельных пациентов могли как повышаться, так и снижаться, в т.ч. при экспрессии соответствующего маркера в ткани опухоли. Показано, что CART экспрессировался только в опухоли (в 4/10 случаев), а MTNR1b и рГПП1 экспрессировались в опухоли (в 6/10 и 10/10 соответственно) и островках Лангерганса (в 5/9 и 9/9 соответственно). Связи экспрессии маркеров с агрессивностью течения инсулиномы не выявлено.ЗАКЛЮЧЕНИЕ. Маркеры CART, MTNR1b и рГПП1 представляют первостепенный интерес для дальнейшего изучения на большей выборке пациентов с инсулиномой. Другие маркеры (TPD52, ХрВ, NESP55, мелатонин, AA-NAT) связь с инсулин-продуцирующей опухолью не показали, поэтому не являются перспективными в отношении будущих изысканий. При этом необходимо продолжать исследования, направленные на поиск новых как циркулирующих, так и ИГХ маркеров, с целью ранней диагностики манифестации заболевания и его рецидива, более точного определения злокачественного и пролиферативного потенциала опухоли.

## Abstract (en)

BACKGROUND: Insulinoma is a neuroendocrine tumor, the main manifestation of which is hypoglycemia. However, the symptoms of hypoglycemia can be non-specific for a long time, especially outside provocative conditions, and quite often the tumor manifests from a life-threatening condition — hypoglycemic coma. In this regard, timely laboratory diagnosis of insulinoma and determination of its aggressive course is one of the priorities in modern researches.AIM: Search for new immunohistochemical (IHC) and circulating markers (CM) of insulinoma, including its aggressive course.MATERIALS AND METHODS: The patients examined at the Endocrinology Research Centre in the period 2017–2022 and operated on for an insulin-producing tumor were included. Before surgery and 2–12 months after it, blood sampling was performed with the determination of targeted marker proteins. Some patients underwent an extended IHC examination of the tumor, surrounding tissue and islets of Langerhans with primary antibodies to target marker proteins with an assessment of the degree of their expression. To determine the aggressive course of the tumor, the degree of malignancy (Grade), the number of tumors and signs of recurrence were characterized.RESULTS: Based on the analysis of literature and pathogenetic characteristics of insulinoma, the following candidates for targeted marker proteins were selected: cocaine and amphetamine-regulated transcript (CART), chromogranin B (CrB), neuroendocrine secretory protein 55 (NESP55), glucagon-like peptide 1 (GLP1), arylalkylamine-N-acetyltransferase (AA-NAT), melatonin, and, exclusively for IHC research, protein D52 (TPD52), as well as receptors for glucagon-like peptide-1 (rGLP1) and melatonin (MTNR1b). 41 patients were included in the study, of which 10 patients underwent an extended IHC study. In patients with both aggressive and non-aggressive insulinoma after surgical treatment, CM levels did not change significantly and in individual patients they could both increase and decrease, including those patients with the expression of the corresponding marker in tumor tissue. It was shown that CART was expressed only in the tumor (in 4/10 of cases), while MTNR1b and rGLP1 were expressed in the tumor (in 6/10 and 10/10, respectively) and the islets of Langerhans (in 5/9 and 9/9, respectively). The association of marker expression with the aggressiveness of the course of insulinoma has not been revealed.CONCLUSION. The markers CART, MTNR1b and rGLP1 are of primary interest for further study in a larger sample of patients with insulinoma. Other markers (TPD52, XgB, NESP55, melatonin, AA-NAT) have not been shown to be associated with an insulin-producing tumor, therefore they are not promising for future researches. At the same time, it is necessary to continue research aimed at finding new both circulating and IHC markers in order to early diagnose the manifestation of the disease and its recurrence, and more accurately determine the malignant and proliferative potential of the tumor.

## ВВЕДЕНИЕ

Диагностика органического гиперинсулинизма у взрослых остается одной из наиболее трудных задач в практической эндокринологии. По данным многочисленных исследований, в том числе отечественных авторов [1][2], вариабельность клинических проявлений, а также дискордантность результатов топической диагностики приводят к поздней диагностике заболевания. В качестве вариантов совершенствования выявления инсулиномы предлагается комбинирование стандартных визуализирующих процедур [1], применение методов молекулярной визуализации [3], построение диагностических моделей на основе анализа панели генов-кандидатов [4]. Эти новые подходы, безусловно, являются наиболее перспективными для практического применения и, как следствие, наиболее обсуждаемыми в научной литературе последних лет. Однако поиск других, в том числе менее затратных методов, продолжается.

В настоящее время в свете выявления новых клеточных сигнальных путей [5][6] проводится активный поиск альтернативных иммуногистохимических (ИГХ) и циркулирующих маркеров (ЦМ) инсулиномы с целью определения злокачественного потенциала, прогнозирования рисков, изучения механизмов опухолевого роста и гормональной гиперсекреции. На основании полученных результатов также могут быть предложены молекулярные мишени для визуализации и лечения.

При анализе последних научных публикаций перспективными в качестве альтернативного маркера нейроэндокринных опухолей рассматриваются несколько кандидатов. Таким может являться опухолевый маркер кокаин- и амфетаминрегулируемый транскрипт (CART) [6], который экспрессируется в нейронах и нейроэндокринных клетках, а также был обнаружен в ткани феохромоцитомы, глюкагономы и инсулиномы [7][8]. CART увеличивает глюкозостимулированную секрецию инсулина in vivo у мышей и in vitro у человека, а также оказывает протективное действие в отношении бета-клеток против глюкотоксичности in vitro у крыс [9]. Есть предположительные данные, что высокодифференцированные инсулиномы, в отличие от менее дифференцированных, характеризуются высоким уровнем иммунореактивности к CART наравне с инсулином [10].

В качестве альтернативного онкомаркера при инсулиноме также может рассматриваться хромогранин В (ХрВ) [11][12]. Преимуществом ХрВ по сравнению с Хромогранином А является отсутствие влияния на его показатели нарушения функции почек и приема ингибиторов протонной помпы [12]. Согласно результатам отечественного исследования [9], частота повышения ХрВ при панкреатических нейроэндокринных опухолях (П-НЭО) составляет 68%. В отличие от Хромогранина А, значимых отличий в уровне ХрВ у пациентов с распространенными (с метастазами в печень) и локализованными формами заболевания не выявлено, что позволяет рассматривать его как универсальный и независимый маркер. Примечательно, что при нормальных показателях Хромогранина А у пациентов с П-НЭО и НЭО желудка в 53,6% случаев наблюдалось повышение концентрации ХрВ относительно порогового уровня [14].

Еще одним возможным биомаркером инсулиномы может быть NESP55 (нейроэндокринный секреторный протеин 55), который обнаружен в островках здоровой ткани поджелудочной железы (ПЖ), хромаффинных клетках, гипофизе и ткани феохромоцитомы, нейробластомы, инсулиномы и других П-НЭО. Согласно результатам отечественного исследования [15], иммунореактивность к данному протеину выявлена в 90,9% П-НЭО.

Предполагается, что в ткани низкодифференцированной инсулин-продуцирующей опухоли (О) ПЖ в процессе неопластической трансформации происходит потеря экспрессии протеина D52 (TPD52), который при иммуноокрашивании обнаруживается в островках Лангерганса (ОЛ) здоровой ткани ПЖ. При этом низкий уровень экспрессии TPD52 ассоциирован с агрессивным клиническим течением инсулиномы, уменьшением безрецидивной выживаемости и выживаемости, связанной с болезнью [16].

С целью уточнения механизмов канцерогенеза, а также определения молекулярных мишеней для визуализации О или радиотерапии большое значение имеет изучение экспрессии различных рецепторов в ткани инсулиномы. В частности, с учетом физиологической экспрессии в эндокринной части ПЖ рецепторов к глюкагоноподобному пептиду-1 (рГПП1), которые обнаружены и в клетках инсулиномы [17–19], преимущественно высокодифференцированной [17][19], разработаны соответствующие методы визуализации: ПЭТ/КТ и ОФЭКТ/КТ с радиотрейсерами на основе аналогов ГПП1. По данным литературы, инсулиномы могут секретировать ГПП-1 [20], однако оценка уровня циркулирующего гормона до и после хирургического лечения опухоли ранее не проводилась.

Кроме того, в клетках инсулиномы выявлены рецепторы к мелатонину MTNR1a и MTNR1b. В недавно проведенном исследовании [21] продемонстрировано, что в наномолярных концентрациях мелатонин стимулирует секрецию инсулина клетками инсулиномы крыс, воздействуя на MTNR1a и MTNR1b, однако при продолжительном воздействии — ингибирует, оказывая дозозависимый эффект. На животных моделях доказано ингибирующее влияние мелатонина на секрецию инсулина посредством воздействия на рецепторы MTNR1a [22], а также на транскрипцию гена инсулина посредством воздействия на рецепторы MTNR1b [23] инсулиномы. Как известно, в норме инсулин и мелатонин ингибируют секрецию друг друга [24]; а при сахарном диабете нарушается циркадный ритм секреции мелатонина [25], что, предположительно, обусловлено изменением активности фермента арилалкиламин-N-ацетилтрансферазы (AA-NAT), катализирующего синтез данного вещества [24]. Однако уровень мелатонина при инсулиноме не исследовался. Таким образом, предполагается, что изучение экспрессии рецепторов MTNR1a и MTNR1b с оценкой показателей мелатонина и фермента AA-NAT позволит выявить новые патогенетические механизмы влияния на функцию опухолевых бета-клеток, что впоследствии приведет к разработке новых терапевтических мишеней при данной О.

Целью настоящего исследования является поиск новых ИГХ и циркулирующих маркеров (ЦМ) инсулиномы, в том числе ее агрессивного течения.

## МАТЕРИАЛЫ И МЕТОДЫ

Критерии включения: пол: мужской или женский. Возраст: 18 лет и старше. Диагноз: инсулин-продуцирующая опухоль (коды МКБ: С25.0–С25.4, С25.7–С25.8), выполнение хирургического вмешательства.

Критерии исключения: не применялись.

Источник случаев: пациенты, обследованные в ФГБУ «НМИЦ эндокринологии» (НМИЦЭ) в период 2017–2022 гг.

Способ формирования выборки: сплошной.

Диагноз инсулиномы подтвержден результатами гистологического и ИГХ исследований. Рецидивом инсулиномы после операции с положительным исходом считается выявление гиперинсулинемической гипогликемии в сочетании со структурными изменениями ПЖ по данным визуализирующих исследований. При этом в качестве положительного исхода хирургического лечения рассматривалась ремиссия гипогликемического синдрома в раннем послеоперационном периоде по данным представленной медицинской документации. В качестве структурных изменений ПЖ, подтверждающих рецидив инсулиномы, рассматривались как новообразование в месте резекции1, так и новообразование другой локализации в рамках первично-множественного поражения2.

Инсулинома считалась агрессивной, если определялись промежуточная степень злокачественности G2 и выше [26] и/или первично-множественная инсулинома (число опухолей >1) любой степени злокачественности и/или рецидивирующая любой степени злокачественности3.

Дизайны исследований:

1)серия случаев с двухкратным наблюдением (до операции и при повторном визите через 2–12 месяцев после операции);

2)сравнительное (группы агрессивной и неагрессивной инсулиномы).

Исследование одобрено локальным этическим комитетом НМИЦЭ (протокол №1 от 27.01.2016 г.).

## Методы исследования

Перед операцией и на повторном визите после хирургического вмешательства всем пациентам выполнен забор крови в пробирки с активатором свертывания в одинаковых условиях искусственного освещения после 8–12 часов ночного голодания. Из венозной крови отделена сыворотка, заморожена и сохранена при температуре -80 °С.

Сбор серологического материала выполнялся исключительно в НМИЦЭ.

Сбор послеоперационного гистологического материала выполнялся в НМИЦЭ и в других медучреждениях, где выполнялось оперативное лечение.

ИФА исследование

Проведено исследование следующих циркулирующих маркеров: CART, ХрВ, NESP55, мелатонин, AA-NAT и у части пациентов (по техническим причинам) ГПП1.

Определение уровня мелатонина проводили методом высокоэффективной жидкостной хроматографии с тандемным масс-спектрометрическим детектированием (ВЭЖХ-МС/МС) на жидкостном хроматографе Agilent 1290 InfinityII (Agilent Technologies, Германия) и тандемном масс-спектрометре AB Sciex QTrap 5500 (AB Sciex, Сингапур) c источником ионизации TurboV (APCI).

ИФА и ВЭЖХ-МС/МС выполнялись в клинико-диагностической лаборатории НМИЦЭ.

Патоморфологическое и стандартное ИГХ исследования

Проводилась оценка числа инсулин-продуцирующих опухолей и их размера. При наличии нескольких опухолей приводилась характеристика наибольшей опухоли. Число митозов рассчитывалось на 10 полей зрения с объективом 40/0.65. В рамках стандартного ИГХ исследования производилось определение индекса пролиферативной активности (Ki67). Стадирование опухолей проводилось в соответствии с классификациями AJCC 2010 [27], ENETS/WHO 2010 [26]. Исследования выполнялись в отделе фундаментальной патоморфологии НМИЦЭ и других медучреждениях.

Расширенное ИГХ исследование

Выполнялось исследование О, окружающей ткани (ОТ) и островков Лангерганса (ОЛ) с первичными антителами к следующим белкам-маркерам: CART, ХрВ, NESP55, рГПП1, MTNR1b, TPD52. Исследованы образцы сыворотки некоторых (произвольно отобранных) пациентов до и после операции, выполненной в НМИЦЭ. Число образцов ткани для анализа было ограничено объемом имеющегося расходного материала. У одного пациента образцы ОТ и ОЛ отсутствовали для анализа в связи с исходным недостаточным объемом послеоперационного материала.

Исследование выполнялось в отделе фундаментальной патоморфологии НМИЦЭ.

Для проведения ИГХ исследования операционный материал фиксировали в забуференном формалине в течение 24 часов, проводили в изопропиловой гистологической проводке, заливали в парафин по стандартной методике. Иммуногистохимическое исследование проводилось на срезах толщиной 3 мкм, расположенных на стеклах с полилизиновым слоем (Leica, Германия) на полностью автоматизированном иммуногистостейнере LeicaBond III (Германия), позволяющем депарафинизировать срезы, проводить инкубацию с антителами при постоянной заданной температуре, проводить энзиматическую демаскировку антигенов, высокотемпературную демаскировку антигенов в буферах pH 6,0 и 8,8, подкрашивать препараты гематоксилином. Исследование проводилось по стандартным протоколам, рекомендованным фирмой-производителем с антителами, представленными выше. Все препараты были отсканированы на сканирующей системе LeicaAT2 (Германия) — уникальной системе получения высокоточных изображений гистологических препаратов, позволяющей создавать полноценное изображение, которое можно многократно увеличивать, просматривая отдельные фрагменты в высоком качестве. Программная обработка изображения позволяет проводить качественный и количественный анализ препарата.

С целью оценки специфичности взаимодействия антител с тканевыми антигенами использовали контрольные образцы: ткани надпочечника (для CART, ХрВ, NESP55), щитовидной железы (для MTNR1a4, MTNR1b, TPD52), ПЖ (для рецепторов к ГПП1), гипофиза (для ХрВ), предстательной железы (для TPD52). Интенсивность окрашивания описывали в соответствии со следующей системой: «−» (0 баллов) при отсутствии иммунореактивности, «+» (1 балл) при слабоположительной реакции (низкоинтенсивное), «++» (2 балла) при умеренно положительной реакции (умеренно интенсивное) и «+++» (3 балла) при выраженной положительной реакции иммуноокрашивания (высокоинтенсивное).

## Статистический анализ

Статистическая обработка полученных результатов проводилась с использованием пакета STATISTICA v. 13 (TIBCO Inc., США). Распределения количественных признаков представлены медианами (Me) и интерквартильными интервалами [Q1; Q3], для некоторых признаков — также минимальными и максимальными значениями. Распределения качественных признаков представлены абсолютными и относительными частотами. Для сравнения связанных групп по количественным признакам применялся критерий Вилкоксона, для сравнения независимых групп по качественным признакам — двусторонний точный критерий Фишера (ТКФ2) и критерий Фримена-Холтона (КФХ, http://vassarstats.net/). Критический уровень статистической значимости был принят равным 0,05.

## РЕЗУЛЬТАТЫ

В исследование включен 41 пациент, их характеристика на момент операции и описание удаленных новообразований приведены в таблице 1. 13 пациентов прооперированы в НМИЦЭ, 28 — в других медучреждениях РФ. Все повторные визиты после операции прошли в НМИЦЭ.

**Table:** Таблица 1. Характеристика 41 включенного пациента Сокращения: И — инсулинома; * Me [ Q1; Q3] (min, max); **Данные доступны у 38 пациентов.

Возраст манифестации И, лет	50 [ 33; 56] (16, 71)*
Пол: м/ж (n; %)	10/31; 24/76
Число И у одного пациента, n	1 (1, 5)
Размеры И, мм	15 [ 13; 20] (10, 40) *
Ki-67, %	2 [ 1; 5] (1, 14)*, **
Ki-67≥3% (n; %)	15 (39)**
G1/G2/G3 (n; %)	25/16/0; 61/39/0
Число митозов, n**	1 [ 1; 2] (0, 4) *
Тип И (солитарная/множественная) (n; %)	34/7; 83/17

Солитарная И наблюдалась в 83% случаев, 95% ДИ (68%, 93%), множественная — в 17% (7%, 32%). Доля небольших (до 2 см) опухолей составила 26/41=63%, 95% ДИ (47%; 78%). Низкую злокачественность (G1) имели опухоли у 61%, 95% (45%; 76%) пациентов. Ki-67%≥3 имели 39%, 95% ДИ (24%; 57%) пациентов. Длительность наблюдения после операции варьировала от 2 до 12 мес. У 9 (22%, 95% ДИ (11%; 38%)) пациентов определен рецидив в сроки от 4 до 12 мес. (рис. 1).

Агрессивная И установлена у 23 пациентов, неагрессивная — у 18.

**Figure:**
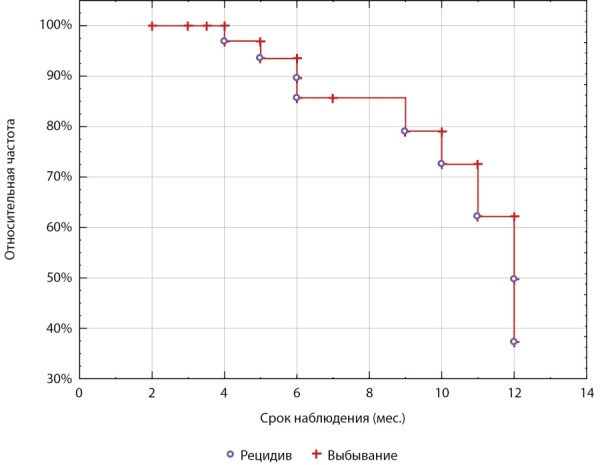
Рисунок 1. Сроки возникновения рецидива в изучаемой группе 41 пациента (кривая Каплана-Майера).

## Циркулирующие маркеры

Проведен сравнительный анализ значений ЦМ до операции и через 2–12 мес после оперативного вмешательства, а также в группах с агрессивной и неагрессивной инсулиномой (табл. 2).

Cтатистически значимых различий не выявлено. Динамика индивидуального уровня всех ЦМ после операции была разнонаправленной — отмечено как снижение, так и повышение показателей.

**Table:** Таблица 2. Циркулирующие маркеры до операции и через 2–12 месяцев после оперативного лечения инсулиномы Сокращения: ЦМ — циркулирующие маркеры; цCART — циркулирующий кокаин- и амефетаминрегулируемый транскрипт, цМелатонин — циркулирующий мелатонин, цХрВ — циркулирующий хромогранин В, цNESP55 — циркулирующий нейроэндокринный секреторный протеин 55, цAA-NAT — циркулирующая арилалкиламин-N-ацетилтрансфераза; цГПП1 — циркулирующий глюкагоноподобный пептид 1 типа. *Ме [ Q1; Q3]; **парный критерий Вилкоксона.

ЦМ	Все пациенты (n=41)	р**	Агрессивная О (n=23)	р**	Неагрессивная О (n=18)	р**
до	после	до	после	до	после
цCART, пг/мл	278,8 [ 240,9; 344,4]*	278,8 [ 229,2; 413,5]	0,464	288,9 [ 240,9; 437,7]	328,8 [ 246,9; 566,8]	0,287	275,4 [ 229,2; 310,1]	257,7 [ 191,3; 317,3]	0,811
цХрВ, нг/мл	65,8 [ 56,9; 83,8]	67,6 [ 55,3; 78,3]	0,403	64,6 [ 56,6; 83,8]	58,4 [ 50,9; 71,7]	0,584	66,6 [ 56,9; 86,4]	76,2 [ 63,5; 91,5]	0,085
цNESP55, нг/мл	0,3 [ 0,3; 4,4]	0,3 [ 0,3; 4,6]	0,808	0,3 [ 0,3; 4,9]	0,3 [ 0,3; 5,5]	0,594	0,5 [ 0,3; 4,1]	0,3 [ 0,3; 2,7]	0,374
цMелатонин, пг/мл	7,0 [ 4,0; 11,5]	7,5 [ 4,6; 14,2]	0,433	7,5 [ 3,7; 14,8]	5,7 [ 4,6; 16,3]	0,761	6,7 [ 4,8; 11,4]	8,1 [ 4,1; 11,9]	0,356
цАA-NAT, нг/мл	0,1 [ 0,1; 0,8]	0,1 [ 0,1; 0,5]	0,459	0,1 [ 0,1; 0,8]	0,1 [ 0,1; 0,7]	0,670	0,1 [ 0,1; 0,8]	0,1 [ 0,1; 0,5]	0,600
цГПП1, пмоль/л	n=27	0,285	n=17	0,130	n=10	0,878
1,5 [ 0,9; 4,5]	1,7 [ 1,1; 2,4]	1,5 [ 1,3; 4,5]	1,6 [ 1,1; 2,2]	1,8 [ 0,5; 3,4]	1,8 [ 1,1; 2,4]

## Иммуногистохимические маркеры

Расширенные ИГХ исследования выполнены у 10 пациентов (табл. 3).

**Table:** Таблица 3. Экспрессия маркеров при иммуногистохимическом исследовании опухоли и окружающей ее ткани, в том числе островков Лангерганса (в баллах) Сокращения: И — инсулинома; Рец+ — наличие признаков рецидива; Рец- — отсутствие признаков рецидива; О — опухоль; ОТ — окружающая ткань; ОЛ — островки Лангерганса; Нет — образец отсутствует, М — митозы.

№ пациента	Характеристика опухоли	Агрессивная И (да/нет)	Локализация	CART	ХрВ	NESP55	TPD52	рГПП1	MTNR1b
1.	Ki67 12%; G 2; n (И)1; рец-	да	О	1	2	1	2	1	1
ОТ	0	3	0	1	0	0
ОЛ	0	3	0	1	2	0
2.	Ki67 8; G 2%; n (И) 1; рец+; n (М) 3	да	О	1	2, неравномерная	1	1	1, неравномерная	1, неравномерная
ОТ	0	1	0	1	0	0
ОЛ	0	2	0	1	2	0
3.	Ki67 <2%; G 1; n (И) 2; рец-	да	О	0	3	1	1	3	1
ОТ	0	3	0	2	0	0
ОЛ	0	3	0	2	2	0
4.	Ki67 <2%; G 1; n (И) 1; рец-	нет	О	0	3	1	2	2	1
ОТ	0	3	1	2	0	0
ОЛ	0	3	1	2	2	1
5.	Ki67 6; G 2%; n (И) 2; рец-	да	О	0	3	1	1	2	1
ОТ	0	3	0	2	0	0
ОЛ	0	3	0	2	2	1
6.	Ki67 <2%; G 1; n (И) 1; рец-; n (М) <2	да	О	1	2	2	3	1	0
ОТ	0	3	1	1	0	0
ОЛ	0	3	1	1	2	0
7.	Ki67 <2%; G 1; n (И) 1; рец-; n (М) <2	да	О	0	2	1	2	1	0
ОТ	0	3	0	2	0	0
ОЛ	0	3	0	2	2	1
8.	Ki67 4%; G 2; n (И) 1; рец+; n (М) 2	да	О	0	2	1	1	2	0
ОТ	0	3	1	2	0	0
ОЛ	0	3	1	2	2	1
9.	Ki67 10%; G 2; n (И) 1; рец-	да	О	0	3	2	2	1	0
ОТ	нет	нет	нет	нет	нет	нет
ОЛ	нет	нет	нет	нет	нет	нет
10.	Ki67 6,4%; G 2; n (И) 1; рец-; n (М) 3	да	О	1	3	1	2	3	1
ОТ	0	3	0	2	0	0
ОЛ	0	3	0	2	2	1

Во всех О зафиксирована экспрессия маркеров: ХрВ (2–3 балла), NESP55 (1–2 балла), TPD52 (1–3 балла), рГПП1 (1–3 балла). Маркеры CART и MTNR1b экспрессировались не во всех О.

Маркер CART не экспрессировался, а NESP55 не всегда экспрессировался в ОТ и в ОЛ, при этом степень экспрессии составляла всегда не более 1 балла. рГПП1 не экспрессировались в ОТ, но всегда в ОЛ (2 балла). MTNR1b не экспрессировались в ОТ, но в ряде случаев выявлялись в ОЛ. Всегда отмечалась экспрессия в ОТ и ОЛ TPD52 и ХрВ: 1–2 балла и 2–3 балла, соответственно. Таким образом, TPD52 и ХрВ экспрессировались везде (в О, ОТ и ОЛ), причем, если для ХрВ более интенсивная экспрессия отмечалась в О, то для TPD52 она не имела каких-то либо особенностей по локализации. Маркер NESP55 в ряде случаев экспрессировался в ОТ и в ОЛ, но всегда в О. Экспрессия CART была ассоциирована только с О (в 4/10 случаев), а MTNR1b и рГПП1 с О (в 6/10 и 10/10 соответственно) и ОЛ (в 5/9 и 9/9 соответственно). Таким образом, CART, MTNR1b и рГПП1экспрессировались в О и ОЛ, но не в ОТ (0%, 95% ДИ (0%; 31%)). Экспрессия CART, MTNR1b и рГПП1 в О представлена на рисунках 2–4.

**Figure:**
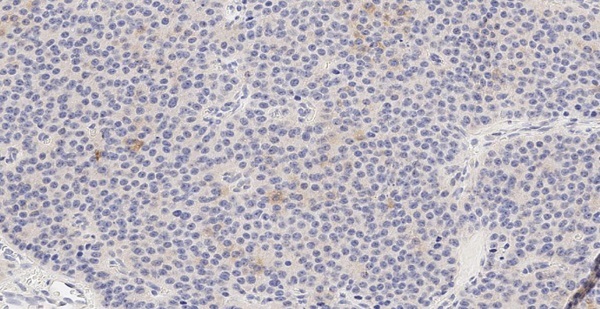
Рисунок 2. Экспрессия в опухоли маркера CART (1 балл).

**Figure:**
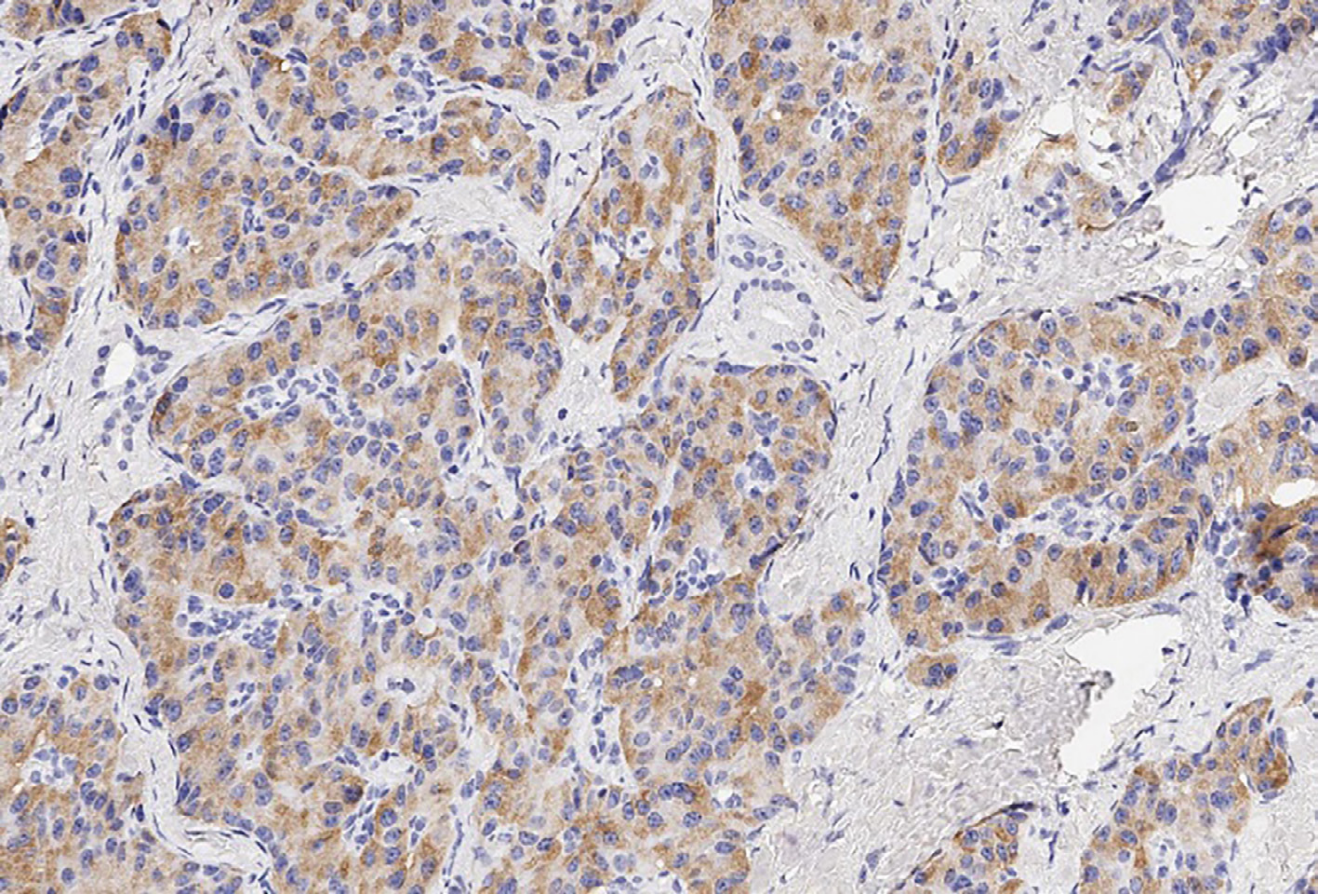
Рисунок 3. Экспрессия в опухоли маркера MTNR1b (1 балл).

**Figure:**
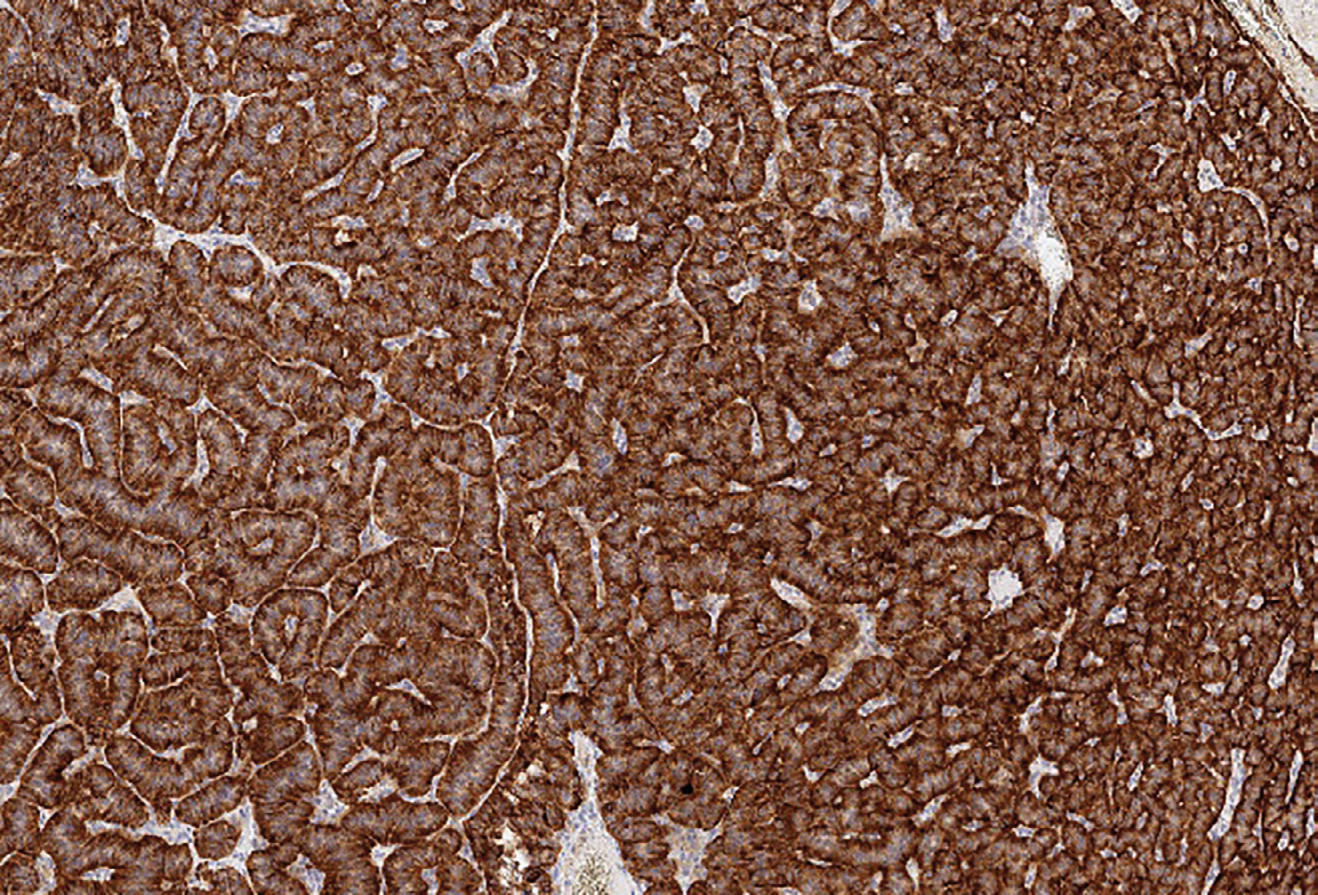
Рисунок 4. Экспрессия в опухоли маркера рГПП1 (3 балла).

При сопоставлении особенностей опухоли с наличием/интенсивностью экспрессии изучаемых маркеров обнаружено, что только при множественной инсулиноме (n=2) маркер NESP55 никогда не экспрессировался в ОТ и ОЛ, а CART никогда не экспрессировался в О; экспрессия маркеров ХрВ в О составляла 3, а экспрессия маркеров ХрВ и TPD52 в ОТ и ОЛ одинаково для каждого маркера составляла 3 и 2 балла соответственно. При рецидивирующей инсулиноме (n=2) экспрессия маркера ХрВ в О составляла 2 балла. При рецидивирующей и множественной инсулиноме экспрессия маркеров NESP55 и TPD52 в О составляла 1 балл.

С учетом полученных результатов проведен поиск взаимосвязи между наличием экспрессии маркера, интенсивностью окрашивания (в баллах), локализацией экспрессии и агрессивностью инсулиномы (табл. 4).

**Table:** Таблица 4. Экспрессия маркеров агрессивного течения заболевания у 10 пациентов с инсулиномой и при разделении на группы с агрессивной и неагрессивной опухолью с учетом степени и локализации экспрессии иммуногистохимического маркера Сокращения: CART — кокаин- и амфетаминрегулируемый транскрипт; MTNR1b — рецепторы к мелатонину МТ2; TPD52 — опухолевый протеин D52; ХрВ — хромогранин В; NESP55 — нейроэндокринный секреторный протеин 55; рГПП1 — рецептор глюкагоноподобного пептида 1 типа; О — опухоль; ОТ — окружающая ткань; ОЛ — островки Лангреганса. * экспрессия в ОТ и ОЛ идентична.

ИГХ маркер	Экспрессия (баллы)	Агресс. инс. (n=7)	Неагресс. инс. (n=3)	Р, ТКФ2
CART, экспрессия в О	0 1	4 3	2 1	1,000
CART, экспрессия в ОТ и ОЛ* (n=9)	0	6	3	1,000
ХрВ, экспрессия в О	2 3	3 4	2 1	1,000
ХрВ, экспрессия в ОТ (n=9)	1 3	1 5	0 3	0,667
ХрВ, экспрессия в ОЛ (n=9)	2 3	1 5	0 3	0,667
NESP55, экспрессия в О	1 2	6 1	2 1	1,000
NESP55, экспрессия в ОТ и ОЛ* (n=9)	0 1	5 1	1 2	0,226
TPD52, экспрессия в О	1 2 3	4 3 0	0 2 1	0,249, КФХ
TPD52, экспрессия в ОТ и ОЛ* (n=9)	1 2	2 4	1 2	1,000
рГПП1, экспрессия в О	1 2 3	3 2 2	2 1 0	0,999, КФХ
рГПП1, экспрессия в ОТ (n=9)	0	6	3	1,000
рГПП1, экспрессия в ОЛ (n=9)	2	6	3	1,000
MTNR1b, экспрессия в О	0 1	2 5	2 1	0,500
MTNR1b, экспрессия в ОТ (n=9)	0	6	3	1,000
MTNR1b, экспрессия в ОЛ (n=9)	0 1	3 3	1 2	1,000

По результатам анализа, значимого предиктора агрессивной инсулиномы не выявлено.

Проведен сравнительный анализ до- и послеоперационного (через 2–11 месяцев) уровня ЦМ у 10 пациентов с экспрессией соответствующего маркера в О (табл. 5).

Изменения уровня ЦМ после проведения хирургического вмешательства не обнаружены.

**Table:** Таблица 5. Циркулирующие маркеры до и после оперативного лечения у пациентов с экспрессией соответствующего маркера в О Сокращения: ЦМ — циркулирующие маркеры; цCART — циркулирующий кокаин- и амфетаминрегулируемый транскрипт; цХрВ — циркулирующий хромогранин В; цNESP55 — циркулирующий нейроэндокринный секреторный протеин 55; цГПП1 — циркулирующий глюкагоноподобный пептид 1 типа; цМелатонин — циркулирующий мелатонин; цАA-NAT — циркулирующая арилалкиламинN-ацетилтрансфераза.

цCART, пг/мл (n=4)	258,2 [ 230,8; 313,8]	227,7 [ 212,6; 241,1]	0,068
цХрВ, нг/мл (n=10)	70,8 [ 59,3; 87,8]	62,7 [ 56,1; 71,7]	0,203
цNESP55, нг/мл (n=10)	1,9 [ 0,3; 12,7]	0,3 [ 0,3; 4,7]	0,173
цMелатонин, пг/мл (n=6)	24,4 [ 11,0; 50,8]	6,6 [ 5,6; 19,0]	0,075
цАA-NAT, нг/мл (n=6)	0,7 [ 0,1; 0,8]	0,1 [ 0,1; 0,5]	0,225
цГПП1, пмоль/л (n=10)	1,7 [ 1,2; 2,5]	1,1 [ 0,6; 1,9]	0,114

## ОБСУЖДЕНИЕ

Полученные результаты согласуются с данными других авторов в отношении возраста манифестации инсулиномы, преобладания пациентов женского пола [28] и медианы размера О [29]. Множественная инсулинома наблюдалась с той же частотой, что и в других исследованиях: 17% (7%; 32%) в нашем исследовании, 10,3% [28] и 8% [29] случаев. В отличие от работ Crippa S et al. [29], а также Iglesias P et al. [28], не отмечалось преобладание О класса G1, однако, в отличие от выборки Wu H. et al. [30], в обследованной нами когорте чаще встречались О с Ki-67 ≥3% (39% против 11,1%). Наиболее вероятно, именно эта особенность обусловливает относительно высокую частоту рецидивов в нашем исследовании (так, в работах Hasanov R. et al. [31] и Crippa S et al. [29] этот показатель составил 14,2% и 3% соответственно). Не исключается также, что более высокая выявляемость рецидивов в нашем исследовании объясняется применением высокочувствительных инструментальных методов диагностики.

Мы впервые оценили возможность применения в качестве опухолевых маркеров CART, ХрВ, NESP55, TPD52, MTNR1b, мелатонина, AA-NAT, рГПП1, цГПП1 исключительно в выборке пациентов с инсулиномой, а не в когорте больных с различными НЭО, как в других работах. При этом изучение большинства маркеров проводилось как в послеоперационном материале, так и в образцах сыворотки до и после операции. Более того, нами впервые исследованы уровни цМелатонина, AA-NAT и цГПП1 у пациентов с инсулиномой.

Значимого ИГХ предиктора агрессивной инсулиномы не выявлено, и это, возможно, связано с малыми выборками в нашем исследовании. Но в случаях, когда у всех пациентов присутствовал только какой-либо один признак, а другой полностью отсутствовал (т.е. результат равнялся нулю), мы позволили себе высказывать предположения о возможном наличии ассоциации этой особенности с агрессивным/неагрессивным течением заболевания. Небольшим числом участников, включенных в исследование, возможно, объясняется и отсутствие значимых отличий в уровне циркулирующих маркеров до и после операции, даже при условии их экспрессии в ткани опухоли.

## CART, цCART

Экспрессия CART в нашем исследовании выявлена только в ткани О и только в некоторых образцах (в 40% случаев — все с солитарной инсулиномой), в отличие от условно здоровых лиц без инсулиномы и больных СД2 в ранее опубликованном исследовании [32], у которых определялась экспрессия CART в ОЛ. Возможно, этот признак является особенностью пациентов с инсулин-продуцирующими О.

Мы показали, что уровень цCARTу пациентов с экспрессией маркера в ткани О значимо не менялся после хирургического лечения, однако у всех пациентов отмечено его снижение, в т.ч. у пациента с рецидивом заболевания. В перспективе при увеличении выборки пациентов с экспрессией CART в ткани О мы предполагаем, что возможно и определение диагностического порога для данного ЦМ. В пользу этого предположения свидетельствуют и данные Bech PR et al. [6], которые выявили повышение цCART в 95% случаев прогрессирующих П-НЭО (всего обследовано 20 больных). Однако, так как авторы не указывают, какие по гормональной активности П-НЭО вошли в выборку, эти данные следует интерпретировать с осторожностью.

## ХрВ, цХрВ

Согласно данным литературы [33], ХрВ играет важную роль в регуляции транспорта секреторных гранул в β-клетках. Потеря его экспрессии приводит к нарушению глюкозозависимой секреции инсулина и процессинга проинсулина с увеличением его высвобождения. В нашей работе, как и в исследованиях других авторов, была подтверждена экспрессия маркера в ОЛ [34], при этом для неагрессивной инсулиномы обнаружена выраженная экспрессия как в ОЛ, так и в ОТ. Данный маркер мы выявили во всех образцах О, но в качестве ЦМ результаты получены неоднозначные. Необходимо отметить, что информация из литературных источников об экспрессии в П-НЭО ХрВ противоречива: так, Sekiya K. et al. [35] обнаружили этот маркер в 4 из 9 образцов инсулином, тогда как Kimuro N. et al. [36] — напротив, не выявили экспрессию ни в одной П-НЭО, включая 8 инсулином. Наиболее вероятно, расхождения в результатах обусловлены использованием различных наборов антител или особенностями конкретных опухолей.

## NESP55, цNESP55

В ранее опубликованных работах сообщалось об экспрессии NESP55 в ОЛ ПЖ [37], а также в ткани инсулиномы, в т.ч. метастатической [38]. Нами подтверждена экспрессия NESP55 в ОЛ ПЖ при солитарной инсулиноме, а также в ткани О. Необходимо отметить, что маркер выявлен во всех образцах О. Однако, в отличие от Jakobsen A-M. et al. [38], в исследованных нами образцах данный маркер также выявлен в ОТ ПЖ при солитарной инсулиноме. Таким образом, в отношении данного маркера также получены неоднозначные результаты.

## TPD52

Как и в работах других авторов, нами подтверждена экспрессия маркера в ОЛ ПЖ, а также ассоциация более низкого уровня (1–2 балла) экспрессии TPD52 в О с ее агрессивным течением [16][26]. Но поскольку маркер экспрессировался и в ОТ, его ассоциация с инсулиномой остается сомнительной.

## рГПП1, цГПП1

Нами также подтверждена экспрессия рГПП1 в ОЛ (не всегда) и в ткани О [39–45]. В обследованной нами когорте обращает на себя внимание отсутствие высокоинтенсивного окрашивания на рГПП1 при неагрессивной инсулиноме. В то же время, согласно данным литературы, экспрессия рГПП1 является признаком преимущественно неагрессивных опухолей, хотя степень экспрессии в этом исследовании не уточняется [17].

Учитывая отсутствие значимого изменения уровня цГПП1 после операции как в изучаемой группе 41 пациента, так и у пациентов с доказанной экспрессией его рецептора в О, можно сделать вывод, что наличие инсулиномы не влияет на секрецию ГПП1.

## MTNR1b, цМелатонин, цAA-NAT

Согласно полученным результатам, экспрессия MTNR1b выявлена нами в 60% образцов О и в 56% образцов ОЛ, что подтверждает данные ранее опубликованных исследований [23][46]. Значимых отличий уровня цМелатонина (как и уровня цАA-NAT) у пациентов с агрессивной и неагрессивной инсулиномой не выявлено. Таким образом, возможно, данный гормон не играет патогенетической роли при инсулиноме.

Таким образом, мы выявили перспективные опухолевые маркеры (CART, MTNR1b и рГПП1), которые требуют дальнейшего изучения. Согласно полученным результатам, прочие маркеры (TPD52, ХрВ, NESP55, мелатонин, AA-NAT) не отличаются селективностью в отношении инсулиномы. В силу малой выборки пациентов, полученные данные носят предположительный характер, необходимо увеличение исследуемой популяции, а также более широкая линейка как потенциальных иммуногистохимических маркеров инсулиномы, так и соответствующих им циркулирующих.

## ЗАКЛЮЧЕНИЕ

Впервые исключительно на выборке пациентов с инсулиномой исследованы маркеры CART, ХрВ, NESP55, TPD52, MTNR1b, мелатонин, рГПП1, цCART, цХрВ, цNESP55, цAA-NAT, цГПП1.

CART экспрессировался только в О, а рГПП1 и MTNR1b — в О и ОЛ, но не в ОТ, в связи с чем именно данные маркеры представляют значительный интерес для дальнейшего изучения на большей выборке пациентов. Другие маркеры не отличались селективностью (TPD52, ХрВ, NESP55 экспрессировались не только в О и ОЛ, но и в ОТ; уровни мелатонина и AA-NAT, как и остальные ЦМ, значимо не менялись у пациентов до и после оперативного вмешательства), что делает их неперспективными в отношении изучения при инсулин-продуцирующей опухоли. Требуется проведение исследований, направленных на поиск новых как циркулирующих, так и ИГХ маркеров, с целью ранней диагностики манифестации заболевания и его рецидива, более точного определения злокачественного и пролиферативного потенциала опухоли.

## ДОПОЛНИТЕЛЬНАЯ ИНФОРМАЦИЯ

Финансирование. Исследование выполнено при поддержке гранта Министерства науки и высшего образования Российской Федерации (соглашение № 075–15–2022–310 от 20.04.2022).

Конфликт интересов. Авторы заявляют об отсутствии явных и потенциальных конфликтов интересов, связанных с содержанием настоящей статьи.

Вклад авторов. Юкина М.Ю. — анализ литературных данных; разработка концепции и дизайна исследования; проведение обследования пациентов; сбор материала; участие в проведении лабораторных исследований; получение, анализ и интерпретация результатов; написание статьи; Трошина Е.А. — помощь в разработке концепции и дизайна исследования; внесение в рукопись существенной (важной) правки с целью повышения научной ценности статьи; одобрение финальной версии рукописи; Урусова Л.С. — помощь в проведении патоморфологических и иммуногистохимических исследований; Нуралиева Н.Ф. — помощь в сборе материала; подготовка статьи к публикации; Иоутси В.А. — помощь в исследовании уровня мелатонина; Никанкина Л.В. — помощь в проведении прочих лабораторных исследований; Реброва О.Ю. — внесение существенной (важной) правки в анализ и интерпретацию результатов; Мокрышева Н.Г. — внесение в рукопись существенной (важной) правки с целью повышения научной ценности статьи, одобрение финальной версии рукописи.

Все авторы одобрили финальную версию статьи перед публикацией, выразили согласие нести ответственность за все аспекты работы, подразумевающую надлежащее изучение и решение вопросов, связанных с точностью или добросовестностью любой части работы.

1. При этом данных за наличие опухоли в крае резекции по результатам патоморфологического исследования после первичной операции не было. В противном случае состояние расценивалось как «продолженный рост опухоли или нерадикальность хирургического вмешательства». 2. В нашем исследовании у всех пациентов рецидив заболевания впервые установлен в ходе повторного визита через 2–12 месяцев после операции. 3. Это не общепринятая схема оценки. Поскольку известно, что даже высокодифференцированная опухоль Grade 1 не может гарантировать благоприятный прогноз и отсутствие метастазирования, мы добавили дополнительные критерии, помимо Grade. Эти дополнительные критерии, по нашему мнению, в большей степени осложняют течение заболевания/препятствуют ремиссии, а метастатической инсулиномы в нашей когорте не было. 4. Экспрессия MTNR1а не подтверждена на контрольных образцах, дальнейшие исследования с данным маркером не проводились.
